# Recent Progress in Nanotechnology for COVID-19 Prevention, Diagnostics and Treatment

**DOI:** 10.3390/nano11071788

**Published:** 2021-07-09

**Authors:** Yousef Rasmi, Kouass Sahbani Saloua, Mahdieh Nemati, Jane Ru Choi

**Affiliations:** 1 Department of Biochemistry, Faculty of Medicine, Urmia University of Medical Sciences, Urmia 5714783734, Iran; rasmiy@umsu.ac.ir; 2 Cellular and Molecular Research Center, Urmia University of Medical Sciences, Urmia 5714783734, Iran; 3 Department of Nuclear Medicine & Radiobiology, Faculty of Medicine, Université de Sherbrooke, Sherbrooke, QC J1H 5N4, Canada; Saloua.Sahbani@usherbrooke.ca; 4 Department of Medical Nanotechnology, Faculty of Advanced Medical Science, Tabriz University of Medical Sciences, Tabriz 5154853431, Iran; nematii.mah@gmail.com; 5 Department of Mechanical Engineering, University of British Columbia, Vancouver, BC V6T 1Z4, Canada; 6 Centre for Blood Research, Life Sciences Centre, University of British Columbia, Vancouver, BC V6T 1Z3, Canada

**Keywords:** COVID-19, nanomaterials, prevention, diagnostics, treatment

## Abstract

The COVID-19 pandemic is currently an unprecedented public health threat. The rapid spread of infections has led to calls for alternative approaches to combat the virus. Nanotechnology is taking root against SARS-CoV-2 through prevention, diagnostics and treatment of infections. In light of the escalating demand for managing the pandemic, a comprehensive review that highlights the role of nanomaterials in the response to the pandemic is highly desirable. This review article comprehensively discusses the use of nanotechnology for COVID-19 based on three main categories: prevention, diagnostics and treatment. We first highlight the use of various nanomaterials including metal nanoparticles, carbon-based nanoparticles and magnetic nanoparticles for COVID-19. We critically review the benefits of nanomaterials along with their applications in personal protective equipment, vaccine development, diagnostic device fabrication and therapeutic approaches. The remaining key challenges and future directions of nanomaterials for COVID-19 are briefly discussed. This review is very informative and helpful in providing guidance for developing nanomaterial-based products to fight against COVID-19.

## 1. Introduction

COVID-19 is an ongoing global pandemic [1,2]. As of May 2021, over 152 million cases have been confirmed with over 3 million deaths. The pandemic has significantly affected the health, safety and well-being of both individuals and communities. Symptoms of COVID-19 range from mild to severe illnesses such as acute respiratory distress syndrome, septic shock or organ failure. Older adults and people with underlying diseases seem to have higher risk of developing life-threatening complications [3]. Since then, significant efforts have been devoted to develop prevention, diagnostics and treatment approaches to combat COVID-19. However, the rapid transmission of the virus along with genetic variation and evolution have dramatically increased global challenges [4,5]. Therefore, in addition to preventive measures, the development of diagnostic tools and vaccines to target the virus or block virus entry have become a priority in the fight against COVID-19 [6]. 

Nanotechnology serves as a powerful tool with the potential to mitigate infections through playing a key role in the prevention, diagnostics and therapeutic strategies for COVID-19 management [7]. These strategies include the development of nanomaterial-based (i) preventive measures and disinfectants, (ii) diagnostic tools for rapid, sensitive and specific diagnostics and (iii) therapeutic agents or vaccines to deliver antiviral agents into human body. In general, nanomaterials such as metal nanoparticles are often less than one micrometer in size, which renders a high surface-to-volume ratio [8]. Other unique characteristics which include improved solubility and multifunctionality enable effective drug delivery, gene modifications as well as optimal interactions between target analyte and capturing molecules on sensors [9]. Hence, nanomaterials have been extensively studied which will likely play a critical role in tackling the current pandemic and mitigating future outbreak.

**Figure 1:**
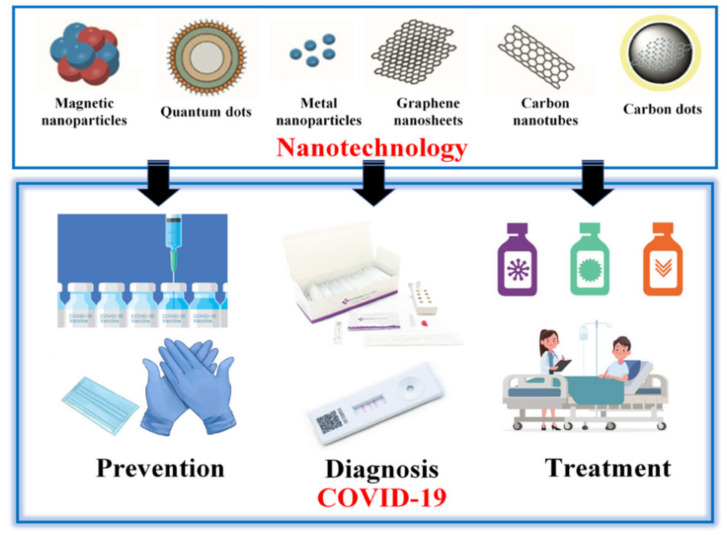
Applications of nanotechnology in COVID-19 prevention, diagnostics and treatment.

This comprehensive review article focuses on the most recent advances in nanotechnology for COVID-19 based on three main categories: prevention, diagnostics and treatment (Table 1, Figure 1), providing comprehensive survey about their usability and performance, which is distinct from the existing review papers where only parts of them were discussed [7,8,9,10,11,12,13]. In this review, we first discuss the use of various nanomaterials such as metal nanoparticles, carbon-based nanoparticles, magnetic nanoparticles and quantum dots for COVID-19. We critically review the advantages of using each nanomaterial along with their use for prevention against the spread COVID-19 (e.g., personal protective equipment and vaccines), rapid diagnostics (e.g., antibody and nucleic acid detection) and therapeutic approaches (e.g., nanodrugs). Finally, the key challenges and future directions of nanotechnology applications for COVID-19 are briefly discussed. Amid the COVID-19 pandemic, this review would be very helpful in providing guidelines for developing nanomaterial-based products to manage the outbreak.

## 2. Prevention

The incidents of COVID-19 outbreaks have increased at an alarming rate. Pandemic prevention strategies involve implementing pharmaceutical (vaccines and antiviral drugs) and non-pharmaceutical countermeasures [21]. As adequate pharmaceutical supplies will not be available instantly, non-pharmaceutical interventions are recommended as a crucial approach [22]. In this regard, the nanotechnology field offers new opportunities to develop strategies for preventing COVID-19 [14]. In this part, we consider the use of nanomaterials (e.g., essentially disinfectants, personal protective devices, and nanocarrier systems) for vaccine development.

### 2.1. Nanomaterials in Masks

Nanomaterials like nanofibres and nanofibre webs are commonly used as a component of masks to minimize large respiratory droplets dispersal and protect staff against droplet transmission by the patient [23]. The filtering face piece containing a combination of a web of polypropylene microfibers and the electrostatic charge is used for high-performance filtering masks (FFP2, FFP3, and N95) [23]. The performance of masks fighting viruses and other microbes has been ameliorating by using filter materials such as nanofibres and nanofibre webs [21] and treating the filter surfaces with materials that have antimicrobial characteristics. The utilization of other nanomaterials such as silver nanoparticles [24], copper oxide [24], copper oxide [25], iodine [26,27], titanium oxide [26] has also been discussed.

Nanofibres offers a vast surface to detain efficiency [27]. They have a tiny void size, low weight, enhanced permeability, and excellent interconnectivity of voids [28]. Chemicals and nucleating agents, including ß-cyclodextrin and o-iodosobenzoic acid have functionalized the nanofibres to decrease the risk of inhaling pathogens and viruses by decomposing or deactivating the contaminants [29]. The standard technique used for the synthesis of nanofiber-based material is electrospinning [30]. Electrospinning produces nanofibers with an electrical charge, enhancing their capability of capturing target particles [31]. Ultrasonic technology has been used for facemask assembly. This technology enables bonds to be rapidly created, producing sealed seams and edges for mask production. It was shown that nanofibre filters include surgical masks led to a lower airflow resistance and enhanced filtration efficiency compared to commercial covers [32]. It was illustrated that the nanofibre filtering facepiece (FFR) possesses a prohibitive pass rate for the fit testing in comparison with the 3M FFRs, and it has a greater bacterial filtration performance compared to other masks in the market [33]. The nanofibre filtering facepiece respirators (FFPR) consisted of partly gelled submicron, nanofibres of polypropylene and a hydrophilic biocide film that could adequately inactivate pathogens [34]. It was found that nanofibre FFPR have excellent air permeability and more powerful antibacterial activities than normal N95 respirators and surgical masks. Therefore, nanomaterial such as nanofiber plays a critical role in improving the functionality of masks.

### 2.2. Nanomaterials in Gloves

In addition to the mask, nanomaterials have also been used in the fabrication of medical gloves for protection against COVID-19 [35]. For instance, some gloves have been developed using silver nanoparticles due to their antibacterial effects [35]. It was confirmed that silver nanoparticles (AgNPs) have virucidal activity. 

Given that COVID-19 viruses penetrate the cells by the angiotensin-converting enzyme 2 (ACE2) receptors, decreasing the levels of ACE2 in the body might aid in reducing the infection rate [36]. It was suggested that capturing the viruses before they entering the cells utilizing nanotechnology in the gloves would be extremely helpful [36]. Nanomaterials comprising ACE2 have been confirmed to be effective in reducing infection rates [37]. Moreover, the ACE2 proteins covered with nanoparticles have shown good catalytic activity and enzyme stability [37] which can be used to produce gloves. These gloves could stop viruses from crossing the host membrane by neutralizing them, hence reducing the spread of the COVID-19.

### 2.3. Nanomaterials in Disinfectants

Nanotechnology offers several opportunities in developing more practical and assuring disinfections. These nanomaterials include metallic nanoparticles (mainly TiO_2_ and AgNPs) and engineered water nanostructures that have anti-viral properties, helping to protect against COVID-19 [14].

Moreover, nanomaterials deliver active compounds in response to photothermal, electrothermal, photocatalytic and others stimuli. Different metallic nanoparticles are also known to have different actions against infections [38]. For instance, AgNPs could be employed as a powerful antibacterial agent. In fact, the bacterial inner and outer cell membranes comprise sulfur- forming proteins and amino acids. Silver interacts with these molecules and inactivates them, which could potentially be applied for SARS-CoV-2. Moreover, particle size and configuration are also other parameters to determine antiviral activity. It was reported that nanoparticles with less than 20 nm show greater attachment to pathogens which causes pathogen death [39]. Hence, they could effectively be used as a disinfectant for COVID-19 [14].

Also, scientists have produced engineered water nano-disinfectants made by several active ingredients, including deionized water, electrolyzed water and hydrogen peroxide solution for microbe inactivation [40]. These nano-sanitizers were examined toward their capacity to efficiently neutralize microbes on both surfaces and air [40]. Their results showed a significant decline in microbe concentration in hydrogen peroxide solution which could be utilized for COVID-19. The Nanotech Surface Company developed a disinfectant formulation containing TiO_2_ and AgNPs, which increases the sterilization effects.

### 2.4. Nanomaterials in Vaccines

Nanoparticles have been extensively investigated for vaccine development due to their tunable size, photothermal and magnetic characteristics, controlled-release properties, and simple functionalization, allowing targeted linking to specific cell types [41]. Many researchers have now utilized them for targeted vaccine delivery to immune cells such as dendritic cells (DCs). Many strategies have been used for selective targeting of DCs with nanomaterials, which show tremendous potential for developing low-dose vaccines [42]. To date, various vaccine candidates have been developed against COVID-19 infection (e.g., subunit vaccines, viral vector vaccines, and DNA vaccines) especially targeting the viral S protein [15]. Nanomaterials have been shown to enhance vaccine potency and immunization strategies to improve immune responses [43].

#### 2.4.1. Gold Nanoparticles 

The physicochemical properties of gold nanoparticles (AuNPs) prevent antibody production against AuNPs, making them an ideal candidate for immunotherapy applications [43]. The AuNPs have been used for virus neutralization. The structural variations in S protein could induce the virus neutralization upon adhering to the AuNP adjuvant [44]. It was shown that AuNPs and S protein connect via electrostatic interactions, and S-protein makes a “protein corona” around AuNPs. A minor modification of AuNP can alter S protein’s immunogenicity in its structure [45]. AuNP-adjuvanted S protein produced an antigen-specific IgG response [45]. The efficacy of adjuvant AuNPs with recombinant S protein against SARS-CoV infection in mice was reported [46].

#### 2.4.2. Ferritin-Based Nanoparticles 

Ferritin-based nanoparticle assembly mediated by RNA presents an ideal candidate for vaccines, allowing the nanoparticles to induce potent and long-lasting antibody responses [47]. For virus-like particles (VLPs), the assembly of non-enveloped viruses is made only by capsid proteins. Ferritins have been used as a platform for the assembly of target antigens. Kim et al. (2018) demonstrated that ferritin-based nanoparticle assembly mediated by RNA could induce CD4^+^ T cell activation, which triggers the production of IFN-γ and TNF-α against MERS-CoV [48]. This approach can potentially be used for COVID-19.

#### 2.4.3. Spike Protein Nanoparticles

Spike protein nanoparticles are commonly utilized as a robust neutralizing immunoglobulin response for SARS-CoV-2 vaccines [49]. The spike proteins have been utilized as a candidate for vaccines as they enhances cellular uptake [49]. These proteins recognize the cell receptors and play an essential role in viral infection [50]. The spike proteins have recently been considered as antiviral drug and vaccine candidates for COVID-19. It is crucial in the protection against re-infection by inducing neutralizing antibodies. The antibodies prevent the virus from being able to enter human cells [50]. Jung et al. (2018) used a recombinant adenovirus serotype to develop an immunogenic vaccine against MERS-CoV [51]. It was reported that the spike protein nanoparticles significantly induced immunoglobulin titers after vaccination of female BALB/c mice [51]. Given that there is a risk of degradation of the proteins after being delivered to patients, the use of nanocarriers can address this challenge for COVID-19 treatment [49].

#### 2.4.4. Hollow Polymeric Nanoparticles

Besides spike protein, hollow polymeric nanoparticles are also known as a candidate vaccine for COVID-19. It was reported that these nanoparticles provoked permanent cellular and humoral immune responses [52]. The hollow polymeric nanocarriers could be prepared by loading a cyclic diguanylate monophosphate covered with receptor-binding domain (RBD) antigens [52]. It was reported that these nanoparticles induced a persistent humoral and T-cell response within the studied mice [52]. Additionally, the mice immunized with these nanoparticle vaccine had an increased RBD-specific IgG2a antibody level without experiencing eosinophilic lung disease [52]. A hollow polymeric or viromimetic nanoparticle-based vaccine coupled with an interferon gene agonist adjuvant against MERS-CoV has been developed, which can also be used for COVID-19 [52].

#### 2.4.5. Lipid Nanoparticles 

**Figure 2:**
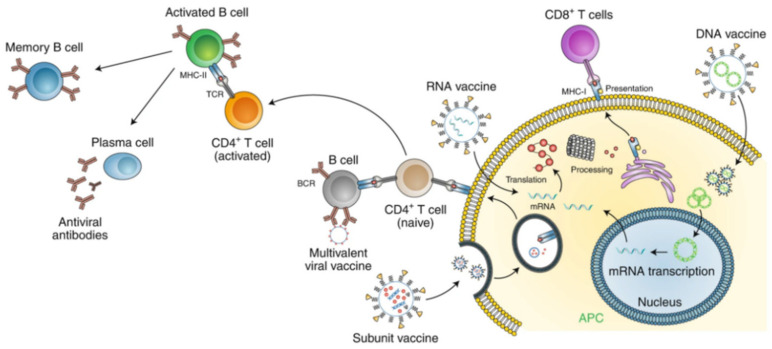
The application of nanoparticle for disease prevention. Lipid nanoparticles are used in nucleic acid-based vaccines. For instance, it has been used in mRNA vaccine development for COVID-19. They act as a carrier to introduce mRNA into host cells and protect mRNA from degradation. Adapted with permission from reference [59] © Springer Nature (2020).

Similarly, lipid-based nanoparticles were used to deliver nucleic acids to target cells (Figure 2). It allows the synthesis of essential viral proteins for vaccination or inactivation of critical viral target genes [47,48]. One vaccination method has been developed by introducing a plasmid containing DNA sequence encoding the antigen(s) into appropriate tissues [53]. These vaccines suffer from inadequate delivery to cells. To address this challenge, nucleic acid-based therapeutic products have been given using a cationic lipid nanoparticle [54]. 

The development of vaccines that involve therapeutic mRNAs has gained considerable attention [55]. Many companies have developed mRNA vaccines encoding SARS-CoV-2 proteins (e.g., spike protein, encapsulated in nanoliposomes) for COVID-19 [56]. These vaccines must be nontoxic and nonimmunogenic [56]. For instance, Moderna developed an mRNA encapsulated in lipid nanoparticles vaccine and performed clinical trials in collaboration with the Vaccine Research Center at the U.S. National Institutes of Health [57]. Pfizer-BioNTech have also developed the similar mRNA-based vaccine which has been authorized for emergency use by FDA to prevent COVID-19.

The absence of immunogenic viral proteins in the lipid nanoparticles makes them more trustworthy than viral vectors. Constituent domains of lipid nanoparticles can be modified to address this challenge [58]. The cationic lipids are nanostructured complexes called “lipoplexes” [58] which have been shown as suitable vehicles in gene therapy, especially for the COVID-19 treatment. The excess cationic coating of these nanostructured complexes could facilitate the binding of nanoparticles with host cells for cytoplasmic transfer of nucleic acids [58].

#### 2.4.6. Protein Nanoparticles

Protein nanoparticles are promising for biomedical applications. They are mainly obtained through recombinant technologies [60]. Scientists used the oligomerization of monomeric proteins method to produce self-assembling protein nanoparticles (SAPNs). It was shown that SAPNs could be engineered to produce a diameter similar to the dimensions of viruses, and hence, it is admitted as a vaccine candidate against respiratory viruses. In general, it evokes an immune response against the respiratory syncytial virus (RSV). The nucleoprotein is the principal target of cytotoxic T-cell response. Nucleoprotein promoter self-assembly was transformed by combining the FsII epitope with a monoclonal neutralizing antibody to the nucleoprotein to create a chimeric nanoring. This assembly improved immune response and protection against RSV [61]. Also, chimeric nanorings were prepared by recombinant technologies. This assembly induced mice protection because these nanorings functionalized with flu virus (A) matrix protein 2 induced local mucosal antibody production when administered via the intranasal route [61]. Because of their similarities with respiratory viruses, these nucleoprotein-nanorings are utilized for intranasal distribution of antigen. Other examples of SAPNs are the coronavirus spike protein and ferritin model and globular nanostructures.

Lipopolysaccharides are components with diameters of 20–200 nm formed by the self-assembly of viral capsid proteins. These particles have the advantage that they can absolutely mimic the structure and the antigenic epitopes of their similar native viruses because they are free from genetic elements [62]. Moreover, this repetitive antigen displays effective phagocytosis by APCs following activation. Recently, it was found that intranasal distribution of influenza-derived VLPs offer protection against different influenza viruses by inducing immune responses [63]. Preparing a stable and spray dried SAPN and VLP vaccines into the intranasal injection can be challenging [64]. The protein nanoparticles have recently been suggested as a potential candidate for the development of COVID-19 vaccines. They have many advantages, including high biocompatibility, stability, molecular specificity, and multivalency to combat COVID-19.

## 3. Diagnostics

Diagnostics plays a key role in the inhibition of COVID-19, which restricts its spread via patient identification and isolation. While a few diagnostics approaches have been introduced, developing a sensitive and rapid COVID-19 diagnostic test is still challenging [65,66,67]. 

Chest computerized tomography (CT) scans and molecular tests have been applied for screening and diagnosing of COVID-19 [68]. Molecular tests (nucleic acid tests) are greater than CT scans for precise diagnoses due to their specific target identifications. Serology test is another approach to assess SARS-CoV-2 infection [69]. Specifically, it can be applied to detect the presence of IgM, IgA and IgG against the SARS-CoV-2 S and N proteins [69,70]. Serological laboratory assays and rapid test formats have been available for COVID-19. While serological tests are simple and effective, they have showed limitations in the diagnostics of acute COVID-19 infections due to the prolonged adaptive immune activation [69]. 

Both nucleic acid and protein diagnostics methods are highly dependent on information such as (1) the genomic and proteomic structure of the pathogen or (2) the alterations in the proteins/gene expression in the host upon infection. As of March 2020, the proteomic and genomic structures of SARS-CoV-2 have been observed, but the host reaction to the virus is still under investigation to enhance these tests for detection of SARS-CoV-2. Various nanomaterials, such as carbon nanotubes, quantum dots, polymeric nanoparticles, metallic nanoparticles, and silica nanoparticles (NPs), are now applied for virus detection [71,72].

### 3.1. Gold Nanoparticles

Gold nanoparticle (AuNPs) is one of the most commonly used nanomaterials for rapid diagnostics [73,74]. For instance, in one study, gold nanoparticle was used to detect double-stranded DNA (dsDNA) of target viruses. In particular, single-stranded DNA (ssDNA) or ssRNA can interact with citrate ions on the AuNP surface, and the addition of salt to the solution can stabilize the particles and change color. In addition, a simple colorimetric hybridization assay was applied to detect dsDNA of SARS-CoV based which is generated from ssRNA. This assay is able to detect target at 4.3 nM in 10 min without requiring any bulky instrument [75]. 

In another study, an effective strategy was developed for immobilizing proteins on the surface of Au using the Au-binding polypeptides. Using the enhanced green fluorescent protein, SARS-CoV-E protein, and core streptavidin of Streptomyces avidinii as models proteins, the Au-binding polypeptide fusion protein was specifically immobilized on AuNP, and the protein nanopatterns on the bare Au surface were demonstrated. These complexes interacts with the antibody, resulting in absorbance and color change, enabling an effective diagnostics of COVID-19 [76]. 

In addition, AuNPs functionalized with green fluorescent proteins showed color and absorbance changes due to interactions with complementary antibodies [77], which could be used for COVID-19. The AuNPs coupled to monoclonal antibodies have also been used as reagents to test another coronavirus, porcine epidemic diarrhea virus (PEDV), in immunochromatographic detection on swine stool samples [77]. One of these methods, disulfide bond-based colorimetric detection, was described to target specific regions of the MERS-CoV genome using thiolated ssDNA probes, to generate a long self-assembled hybrid, which protects citrate ion-coated AuNPs from salt-induced aggregation [78]. It can validate the presence of virus through a localized surface plasmon resonance (LSPR) shift and AuNPs color changes [78]. Immobilization is achieved relatively rapidly by thiol-gold interaction, the response of the gene sensor has been correlated with biotinylated target concentrations between 2.5 and 50 pmol/L, with a detection limit of 2.5 pmol/L [79].

Additionally, an AuNP-based electrochemical hybridization approach was described using a gene sensor, which consists of a thiolated-DNA probe-immobilized on the AuNPs carbon electrode to hybridize biotinylated-target DNA. Immobilization is achieved by thiol-gold interaction, and the sensor response was observed with the target concentration of 2.5–50 pmol/L, with a detection limit of 2.5 pmol/L [79]. Chiral gold nanohybrids (CAuNPs) with quantum dots (QD) were formerly used to develop plasmonic AuNPs with self-assembled star-shaped keys for the detection of other viruses, such as CoV. In this method, each CAuNPs and QDs was electrostatically coupled to two virus-specific antibodies and a nano-sandwich assembly when a specific virus was present, resulting in an excited QD position coupling with excellent plasmonic resonance. The sensitivity of the bioassay was found to be 1 pg mL^−1^ [80]. To diagnose coronavirus target, an electrochemical chip was introduced via a carbon electrode composed of AuNP array [81]. The coronavirus protein was bound on an AuNP-electrode, and both coronavirus protein and free viruses compete for binding sites in the presence of antibodies. There was a good linear response between the sensor response and the concentrations of coronavirus ranging from 0.001 to 100 ng mL^−1^. The assay was performed achieved the detection limit of as low as 1.0 pg mL^−1^. The approach was single-step, sensitive and accurate. It was successfully used to test spiked nasal specimens [81].

In another study, a method was introduced to visually detect COVID-19 virus without using sophisticated instruments. Colorimetric detection was developed using thiol-modified antisense oligonucleotides (ASOs)-coated AuNPs designed specifically for the N genes. Thiol-modified ASO-cap AuNPs were selectively aggregated in the presence of the SARS-CoV-2 target RNA sequence and showed a change in its surface plasmon resonance. The result can be observed in 10 min with a detection limit of 0.18 ng/μL [82]. 

Furthermore, one group developed a lateral flow assay for the rapid detection of IgM against COVID-19 through the indirect immunochromatography approach [83]. Generally, the SARS-CoV-2 nucleoprotein (SARS-CoV-2 NP) was coated on an analytical membrane for target capturing, and anti-human IgM was conjugated to AuNP, serving as a detection reporter. AuNP-LF analysis showed excellent selectivity in the IgM detection without interference from other viruses. Each assay only requires 10–20 μL serum and the result can be obtained within 15 min.

Zhao et al., reported the synthesis of poly (amino ester) with carboxyl groups (PC)-coated magnetic nanoparticles (pcMNPs along with the relevant RNA extraction techniques. Leveraging from the test simplicity, this novel extraction technique can significantly reduce the assay time and simplify the user steps. It combines both lysis and binding steps into one step and the pcMNPs-RNA complexes can be incorporated into subsequent RT-PCR reactions. This test detects two different regions (*ORFlab* and *N* gene) of viral RNA, and the detection limit of 10-copies of SARS-CoV-2 pseudovirus particles was achieved [84].

More recently, the detection of COVID-19 using non-invasive approaches have been proposed [85,86]. One study has demonstrated the detection of COVID-19 biomarkers from exhaled breath using a AuNP-based sensor [86]. The sensor consisted of different AuNP linked to organic ligands as well as inorganic nanomaterial film. The inorganic film is responsible for the electrical conductivity. When exposed to the volatile organic compounds (VOCs) from exhaled breath, the organic film reacts with the VOCs, resulting in the inorganic film swelling or shrinkage as well as the changes in electrical conductivity. This non-invasive sensor could potentially be used for rapid screening of COVID-19.

### 3.2. Magnetic NPs (MNPs)

**Figure 3:**
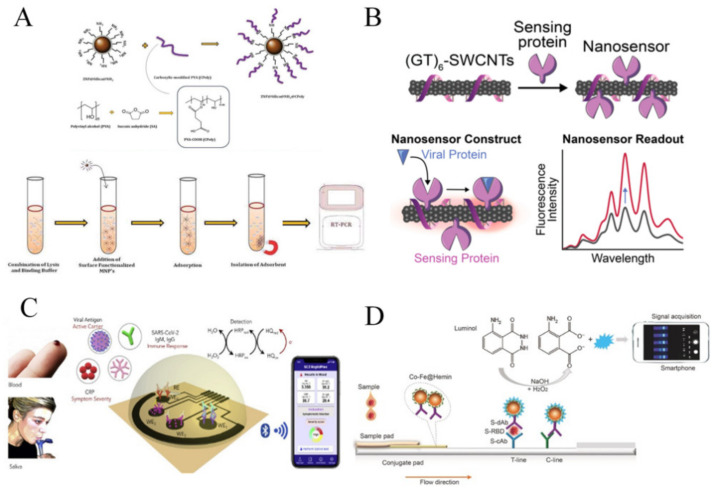
The application of nanoparticles for COVID-19 diagnostics. (**A**) Magnetic particles were used for viral RNA extraction for COVID-19 diagnostics. Adapted with permission from reference [88] Taylor & Francis © (2021). (**B**) The use of a single wall carbon nanotube-based optical sensor for fluorescent detection of COVID-19. Adapted with permission from reference [89] Creative Commons Attribution License © (2021). (**C**) A wireless graphene-based telemedicine platform (SARS-CoV-2 rapidplex) for rapid and multiplex electrochemical detection of SARS-CoV-2 viral proteins, antibodies (IgG and IgM) and inflammatory biomarker c-reactive protein (CRP) in blood and saliva samples. Adapted with permission from reference [90] Elsevier © (2020). (**D**) Development of nanozyme-based chemiluminescent paper-based biosensor for SARS-CoV-2 antigen. Adapted with permission from reference [91] Elsevier © (2021).

Magnetic NPs (MNPs) are commonly used for nucleic acid separation prior to detection [87]. Accurate detection requires efficient extraction and separation of nucleic acids from samples which allows target purification. For example, superparamagnetic nanoparticles (80 nm) conjugated with a probe which is complementary to the target sequence SARS-CoVs was used in one study. Using a magnet, the functionalized superparamagnetic nanoparticles have the potential to extract target cDNA from specimens [16]. The amount of extracted DNA was increased through PCR which was tested using silica-coated fluorescence nanoparticles conjugated with complementary sequence. Silica-coated fluorescence NPs produces fluorescence signals, which is directly correlated to the concentration of the target cDNA [16]. In another study, Somvanshi et al., reported the fabrication of the surface functionalized MNPs and the viral RNA-extraction protocol for potential COVID-19 diagnostics (Figure 3A) [88]. The zinc ferrite nanoparticles were synthesized by combustion, and the nanoparticle surfaces were functionalized with silica and carboxyl-modified polyvinyl alcohol. This platform shows the ability to automatically extract the viral RNA from diverse sample types. It reduces the operation steps, which offers great potential for COVID-19 molecular-level diagnostics.

Further, another study introduced a one-step nucleic acid extraction procedure that specifically binds viral RNA using polycarboxyl-functionalized amino group-modified MNPs (PC-coated NH2-MNP). Nucleic acids were accumulated using magnetic field, and then they were released from the MNPs by the addition of wash buffer [84]. By detecting COVID-19-pseudoviruses, polycarboxyl-functionalized MNPs showed excellent absorption and paramagnetic properties through fast capture (30 s magnetic capture) of targets. 

### 3.3. Quantum Dots

Quantum dots (QDs), also known as “semiconductor nanomaterials” with the sizes of 1–10 nm have been widely used for COVID-19 diagnostics. QDs have been known as a new fluorescent probe for molecular imaging [92]. The unique characteristics of QDs, including their optical properties, have made them a great candidate to serve as a fluorescent label. In addition, their emission wavelength can be easily and precisely tuned by changing their size [93]. Owing to its excellent properties, QDs are now predominant imaging probes (chemosensors and biosensors) for sensing [94]. For instance, Ashiba et al. [95] introduced a highly sensitive biosensor to detect virus and prevent the spread of infections. A surface plasmon resonance (SPR)-assisted fluoroimmunosensor was created and a QD fluorescent dye was used for the assay. The QD excitation efficiency, degree of electric field enhancement by SPR, and intensity of autofluorescence of the substrate on the chip were optimized to reduce the background signals. As the result, the sensor was able to achieve the detection limit of 0.01 ng/mL virus, corresponding to 100 virus particles.

In another study, a QD-conjugated RNA aptamer-based chip was introduced for highly sensitive and rapid detection of SARS-CoV N protein [96]. Specifically, the QD-conjugated RNA aptamer can specifically bind to the SARS-CoV N protein immobilized on the chip, producing an optical signal. The detection limit of as low as 0.1 pg mL^−1^ was achieved [96]. In short, the use of fluorescent-based QDs may assist researchers in developing sensitive diagnostic approaches for COVID-19 [97].

### 3.4. Carbon-Based Nanomaterials

Carbon-based nanomaterials have been broadly used in developing platform for COVID-19 diagnostics. Carbon nanotubes (CNTs), graphene, and carbon dots (CDs) can be categorized as zero-(0D), one-(1D), and two-(2D) dimensional carbon nanomaterials [98]. Carbon dots were discovered in 2004 [99] and they normally have photoluminescence, biocompatibility, and high stability, predisposing them with different applications, including biosensing and bio-imaging [100,101,102].

The use of CNTs for diagnostics of respiratory viruses including SARS-CoV-1 and SARS-CoV-2 have been reported. Yeh et al., [103] reported a novel CNT size-tunable enrichment microdevice (CNT-STEM) that could enrich and concentrate viruses from raw samples. Generally, the channel sidewall in the microdevice was fabricated by nitrogen-doped multiwalled CNTs, where the intertubular distance between CNTs is optimized to match the size of different viruses. Using this device, the avian influenza virus strain was identified. The CNT-STEM significantly enhances virus isolation rates and detection sensitivity [103]. Because of the ease and reliability of this technique, it can be modified to detect SARS-CoV-2 RNA or proteins. In another study, a single wall CNT (SWCNT)-based optical sensing approach was introduced for COVID-19. A nanosensor consisted of SWCNTs that noncovalently functionalized with ACE2 was developed, which presents a high binding affinity for SARS-CoV-2 spike protein. The use of SWCNT resulted in two-fold fluorescence signal increase in the presence of target viruses (Figure 3B) [89].

Moreover, a nanomaterial-based biosensor that could rapidly detect COVID-19 antibodies was developed [104]. The biosensing platform was made using 3D bioprinted electrodes coupled with nanoflakes of reduced-graphene-oxide (rGO). Specific viral antigens were immobilized on the rGO nanoflakes to detect targets. The antibodies were selectively bound to the antigens after being introduced into the device, altering the impedance of the electrical circuit. The detection limit for antibody tests against SARS-CoV-2 spike S1 protein and its receptor-binding-domain (RBD) were 2.8 × 10^−15^ and 16.9 × 10^−15^ M, respectively [104]. In another study, SARS-CoV-2 RapidPlex, a portable, wireless electrochemical platform, was introduced for rapid detection of COVID-19 [90]. It detects viral antigen nucleocapsid protein, IgM and IgG antibodies, and inflammatory biomarker such as C-reactive protein. The platform showed highly sensitive and selective, for the detection of SARS-CoV-2 in blood and saliva samples (Figure 3C) [90]. 

Apart from these, nanodiamonds have received significant attention for COVID-19 diagnostics due to its high stability and low cytotoxicity. In one study, fluorescent nanodiamonds were used as an ultrasensitive label for COVID-19 lateral flow immunoassay [105]. These nanodiamonds were immobilized on the test line, and microwave field was used to selectively separate their fluorescence signal from background signal, which significantly improved the detection sensitivity. This assay was 10^5^ more sensitive than the conventional gold-nanoparticle-based lateral flow assay. Altogether, these studies suggest that the carbon-based nanomaterials can be used as an antiviral therapeutic agent for COVID-19 [106]. 

### 3.5. Nanozymes

Nanozymes are artificial enzymes composed of nanomaterials having the similar efficiency as natural enzymes [107]. Nanozymes have outstanding catalytic activities, fast response and self-assembly capability, which have been broadly used for disease diagnostics and treatment [19,108,109]. For example, a novel nanozyme-based chemiluminescence paper-based biosensor for COVID-19 (Figure 3D) [91]. Traditional chemiluminescence immunodiagnosis uses natural proteases such as HRP or alkaline phosphatase that showed limitations such as low storage stability, complex preparation procedures and high-cost. The proposed biosensor used peroxidase-mimic Co-Fe@hemin nanozyme instead of natural horseradish peroxidase (HRP) that could significantly amplify the chemiluminescent signal, achieving the detection limit of 0.1 ng/mL. The Co-Fe@hemin nanozyme was shown to have a better stability for temperature and acidity or alkalinity as compared to HRP, which can be stably stored at room temperature. Hence, the proposed biosensor can potentially be used for COVID-19 diagnostics at remote and resource-limited settings [91].

## 4. Treatment

The spread of new viruses and their heterogeneity require novel therapies [110]. The main limitations of current antiviral treatment are poor specificity, resulting in host cell cytotoxicity. Nanotechnology creates a new opportunity for antiviral therapy. The flexibility of nanoparticles makes them tunable vectors for specific therapeutic drug delivery and virus targeting. The approach of using nanoparticles to battle SARS-CoV-2 could contain mechanisms that influence the virus entry into the host cell until their inactivation. The inhibition of the viral surface proteins may lead to virus death, so targeting nanoparticles, particular to virus expressed proteins could decrease the viral internalization [111]. Organic nanoparticles have been applied for delivering antiviral drugs such as acyclovir, zidovudine, efavirenz, and dapivirine, to enhance drug bioavailability, drug delivery and targeted antiviral activity [112]. Antimicrobial drugs have been tested in clinical trials for COVID-19, such as lopinavir, chloroquine, remdesivir, ritonavir, and ribavirim and have demonstrated promising results against SARS-CoV-2 [84].

Metal-loaded nanocomposites and metal nanoparticles are known to be highly effective against viruses and microbes due to their distinctive characteristic, and the ability to control the release of ions. For example, the controlled release of metals such as Ag, Fe, Cu, Zn, TiO_2_, CdS and MnS_2_ showed potential antiviral and antimicrobial properties of metal grafted GO [113,114]. Nanotechnology can help in the advancement of COVID-19 drug delivery due to the following advantages: (I) the nanoparticle morphology and size permit the drug delivery to physiologically unavailable locales without inducing reticular endothelial cells immune response [115], (ii) their great surface-to-volume ratio enhances drug loading [116], (iii) the capacity of nanoparticles to cross membranes with negatively charges due to their surface charge alteration [117], and (iv) nanoparticles such as silver (Ag) and AuNPs possess intrinsic virucidal activity [118]. The existing nanoparticles for the CoVs treatment are summarized in Table 1.

### 4.1. Exosomes

Therapeutic safety and efficacy for delivery of exosomes to the target cell has now gained great attention. Several clinical applications have introduced them as potential biological nano-carriers for COVID-19 treatment [119,120,121]. These applications are based on their ability of escaping immune recognition, fast degradation, slightly negative zeta potential for longer transmission time in the body and small size for effective tissue penetration [119,122]. Various approaches can be utilized to develop optional exosomes including (I) indirect engineering approaches and (II) direct engineering approaches [123]. In the indirect engineering approach, some cells such as stem cells are cultured with therapeutic agents or genetically changed to make artificial/pharmaceutical exosomes, while in the direct engineering method, therapeutic agents are loaded directly into exosomes separated from the source cells. These exosomes are then transferred into the target tissue [124]. In fact, there are three stages in their creation from endocytic cellular pathway: (i) endocytic vesicles creation via plasma membrane invagination, (ii) inward budding of the limiting late endosomal membrane that induces multivesicular bodies (MVBs), and (iii) merging of the MVBs with the plasma membrane to form exosomes [125]. 

Evaluation of exosomes as immunogenic factors for the treatment of SARS coronavirus infection has been studied. Exosomes composed of SARS virus coronavirus S protein produce neutral antibody titers that increase with initial vaccine preparation and then with beneficial adenovirus vector vaccine [20]. The scientists determined the use of these synthetic exosomes for treatment. To confirm that the SARS coronavirus S protein was incorporated into exosomes, SARS-S transmembrane domains were replaced by those of the vesicular stomatitis virus G protein to make chimeric protein-functionalized exosomes for application as SARS coronavirus vaccine [20]. Also, to treat SARS-CoV-2 pneumonia, researchers suggested using exosomes as drug delivery systems [120]. 

The exosomes possess hypoimmunogenic properties, making them extremely stable to migrate to the target organ for immunotherapy. These extracellular vesicles are known to be responsible for the transfer of genetic material from stem cells to the target cell for regeneration [119,126]. Today, this approach has been used to treat COVID-19. The combinatorial strategies of antiviral drugs and stem cells with high capabilities of immunomodulation, tissue protection and healing along with their exosomes could reduce the severity of the COVID-19 [119].

### 4.2. Metal Nanoparticles

Metal nanoparticles such as gold nanoparticles (AuNP) and silver nanoparticles (AgNPs) are vastly examined nanotechnology method to treat viral infections. Several hypothesis of nanoparticles have been prepared to progress a new strategy to improve or eliminate the infection severity [127]. In recent research, it determined that colloidal Ag with particle sizes between 3–7 nm can be very effective to treat and prevent viral infection at early stage of respiratory infections [127]. Sarkar and coworkers has hypothesized that the use of water dispersed AgNPs (10 nm) in combination with bronchodilators in lungs via bi-level ventilation or simple nebulizer machine may induce better virucidal action [128]. AgNPs (30 nm) on the magnetic hybrid colloid (comprising amine-functionalized SiO_2_-Fe_3_O_4_ particles) display a promising nanosystem for inactivating virus. The systems have potential for interaction with virus proteins by linking among the thiol groups and Ag ions. In addition, Ag ions may produce ROS for the virus inactivation [129].

Anti-SARS-CoV-2 activity was only observed with AgNPs of diameters ranging from 2 to 15 nm. Immunofluorescence study confirmed that polyvinylpyrrolidone capped 10 nm silver nanoparticles (PVP-AgNP10) completely inhibited SARS-CoV-2, but AgNP100 did not [130,131]. Various inhalable and ingestible formulations of AgNPs are available as therapeutic agents in the market [131,132]. They can be used on a diversity of inanimate surfaces to reduce the spread of COVID-19 [131]. AuNPs have also shown potential for vaccine development as they could induce immune response via internalization by APC [133,134]. 

### 4.3. Metal Oxide Nanoparticles (MONPs)

The antimicrobial activity of metal oxide nanoparticles (MONP) was recently studied [135,136]. Numerous mechanisms of action have made MONPs an efficient antimicrobial agent and the main mechanism is related to ROS production. ROS oxidizes several biomolecules and sites of microorganisms which results in cell death [137]. Several viral strains have been found to be resistant to the recent therapeutic methods used by metal oxide nanoparticles. Therefore, the therapeutic potential of MONPs have been further investigated.

For example, iron oxides nanoparticle (IONPs) antimicrobial activity has been frequently studied [135,136] against influenza virus (H1N1) [129], dengue virus [138] and rotavirus [139]. IONPs are FDA accepted and biocompatible for treatment of anemia [140]. It was hypothesized that IONP interact with the proteins of virus surface and interference with virus attachment and/or entry into the host cell, inducing neutralization. Results showed that IONPs could be a hopeful candidate to be either for infection prevention and control or as antiviral agent. So, IONPs could be a harmless and promising candidate for fast use in the COVID-19 patient therapy. Other iron oxide particles like ZnO nanoparticles (ZnO NPs) are characterized by non-cytotoxicity, biocompatibility and availability. There are a few studies on the antiviral action of ZnO NPs [141,142]. One study examined the antiviral activity against H1N1 and showed that polyethylene glycol (PEG) coated ZnO-NPs (PEG-ZnO NPs) had higher antiviral activity and lower cytotoxicity than “naked” ZnO NPs. Therefore, ZnO NPs may act as an effective antiviral nanomaterial for COVID-19 treatment [18,142,143].

Additionally, functionalized metal nanoparticles act as antiviral agents by blocking the virus attachments and entry into the host cells [142,144]. Specifically, supra-magnetic iron oxide nanoparticles (SPIONPs) serve as magnetic anchors to target molecules as well as contrast agents for MRI [142,145]. Lipid-coated SPIONPs are able to deliver antiviral agents to targets of interest [142,146]. The antiviral potential of these nanoparticles can be attributed to their adsorption on viral surfaces and subsequent local changes such as “glycoprotein agglutination”, thereby preventing virus penetration and entry into host cells [142,147,148]. Therefore, they offer tremendous potential for treatment of COVID-19.

### 4.4. Carbon-Based Nanomaterials

The antiviral activity of promising nanoplatforms has increased the use of GO and its derivatives against viral infections. According to reports, composite structure and charges significantly influence the antiviral effects. It has also been revealed that GO prevents viral infections through virus inactivation prior to virus entry into host cells. Generally, the negatively charged GO might interact with positively charged viral lipid membrane inducing its rupture. Some parts of spikes and envelope were destructed after incubation of GO with virus [149]. Silver graphene nanocomposites block the coronavirus in a concentration-dependent manner against enveloped and non-enveloped viruses. This type of nanocomposite repressed the both non-enveloped and enveloped viruses infectivity, and it has greater coronavirus inhibition than GO [114].

Carbon nanocomposites can be functionalized by binding to polymeric or metallic NPs through surface functional groups such as lactones, carboxylic acids, and hydroxyls [150]. Activated carbon plays an important role in limiting the recent spread of COVID-19, which is caused by an increase in its ability to retain viruses. Conventional powdered activated carbons remove viral particles by entrapment in their nanopores through a hydrophobic interaction with the surface of the virus [151].

There are particular instances of viral particle interaction with various kinds of carbonaceous nanomaterials such as carbon quantum dots (CQDs), nanodiamonds (ND), activated carbons, SWCNT or multiwall carbon nanotubes (MWCNT), GO, and graphene. Their potential applications in COVID-19 treatment are the elimination of viral particles from water or air through various virucidal mechanisms [150]. 

Carbon quantum dots (CQDs) are important options for interacting with viruses and preventing viruses from entering host cells. Recently, it was shown that functional CQDs with boronic acid ligands interfered with the function of the S-coronavirus protein and significantly inhibited its entry into host cells. The study outcome showed that the addition of these nanomaterials to cell culture media, before and during coronavirus infection, significantly reduced the rate of cell infection [17,152]. In addition, functionalization of ribavirin or isoprinosine on the SWCNT surface has improved drug performance. CNT-based nanosystems are promising to modify viral genomes and reduce viral activity [153,154]. CNTs with wide capacities for targeted delivery of diverse theranostics could be utilized as antiviral agent nanocarriers in COVID-19 therapy [155,156,157,158,159].

### 4.5. Quantum Dots (QDs)

QDs are flexible semiconductor particles that have the ability to emit photons emitting with particular wavelengths which provides high-sensitive and strong fluorescence for POC viral assay [160]. Functional carbon quantum dots (CQDs) may serve as therapeutic mediators for human coronavirus. Following internalization and interaction with S protein, the virus function would be inhibited by these nanoparticles in a nanoparticle concentration-dependent manner [17]. In recent years, curcumin-based cationic CDs have been produced by hydrothermal techniques to fight against the coronavirus [161].

**Figure 4:**
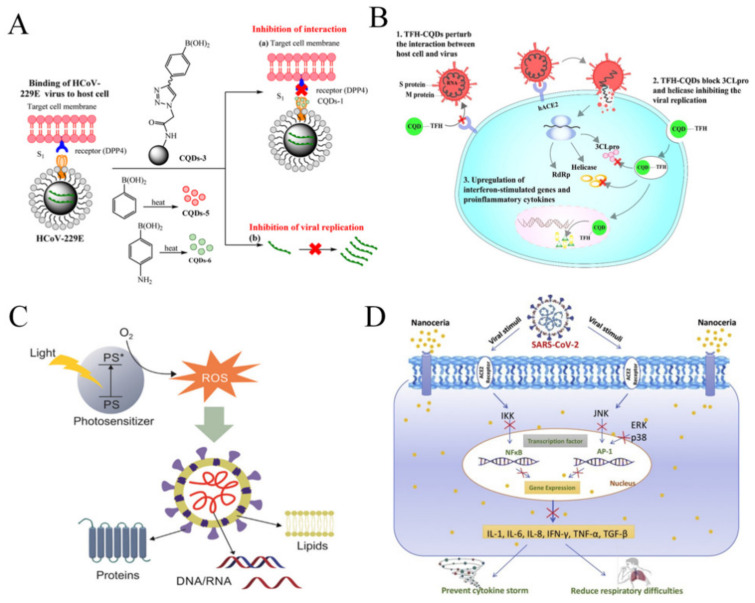
The application of nanoparticles for potential COVID-19 treatment. (**A**) Carbon quantum dots inhibit binding of S protein receptor of coronavirus to host cells and prevent viral RNA genome amplification. Adapted with permission from reference [106] © Creative Commons Attribution License (2020). (**B**) Triazole-based carbon quantum dots were suggested for use as an antiviral agent to treat COVID-19. Adapted with permission from reference [162] © Elsevier (2020). (**C**) Semiconductor nanoparticles, including quantum dots can generate antiviral radicals by interacting with light. Adapted with permission from reference [9] © Creative Commons Attribution License (2020). (**D**) Nanoceria inhibits cytokine storm due to its strong anti-inflammatory effects, serving as a therapeutic agent for COVID-19. Adapted with permission from reference [163] © Creative Commons Attribution License (2020).

QDs play an important role for the treatment of human CoVs infections. For instance, in one study, the antiviral function of seven different CQDs for the treatment of human CoV HCoV-229E were studied. The CQDs, with a size about 10 nm have significant solubility in water, which were generated using hydrothermal carbonation of carbon precursors, ethylenediamine/citric acid, and post-synthetic modification using boronic acids. Concentration-dependent virus inactivation was revealed upon the QD treatment [17]. The blockage of the HCoV-229E entry into host cells is possibly due to the interaction of the CQDs functional groups with the HCoV-229E entry receptors. In addition, the viral replication phase was also inhibited (Figure 4A) [106].

In another study, triazole-based CQDs were suggested for use as an antiviral agent to treat COVID-19 (Figure 4B) [162]. CQDs consist of hydrophilic functional groups that make them suitable for various biomedical applications. They serve as a multi-site inhibitor by blocking the viral entry, their RNA synthesis and replication [161]. Their concentration is essential for regulating the number of viruses in the body [162,164]. In addition, the exposure to the virus, the accumulation of reactive oxygen species expressed in cells at the same time by resetting the expression of pro-inflammatory cytokines is reduced. It has also been reported that CQDs synthesized from glycyrrhizic acid exhibit a strong antiviral behavior towards RNA virus [162,165]. It was expected that CQDs or their functional analogues exhibit strong virucidal activity rather than only blocking entry of coronaviruses or human RNA viruses into host cells. The varying responsiveness of some viruses suggests that CQDs also differ in antiviral strategies and require further evaluation [162].

In addition, semiconductor nanoparticles, including QDs can generate antiviral radicals by interacting with light. This process is usually referred as virus and other microbial photodynamic inhibitors (PDIs) and is activated by both organic photosensitizing (PS) and inorganic semi-conducting nanoparticles compounds [166]. A general aspect in both groups is the light-induced production of ROS [167]. These interactions may potentially destroy viral components like the membrane, DNA/RNA, and protein (Figure 4C) [9].

### 4.6. Drugs and Chemical Compounds

#### 4.6.1. Peptide Inhibitors

Peptide inhibitors have also been used for the treatment of COVID-19. In one study, heptad repeat 1 (HR1) peptide inhibitors were utilized as an inhibitor for HR1/HR2-mediated sheath merging between MERS-CoV and host cells, the main conduit for host infections induced by MERS-CoV [80]. Peptide inhibitors against coronavirus SARS-CoV-2 have been studied. These inhibitors are often produced by two consecutive α-helixes (bundles) derived from the protease domain (PD) of the ACE2 that binds to the binding domains of SARS-CoV-2 receptors. Molecular dynamics simulations have shown that the α-helical peptide retains its secondary structure and provides a very specific and stable binding to block SARS-CoV-2 from entering the host cells. Many of these peptides can bind to nanoparticle carrier surfaces to establish polyvalent binding to SARS-CoV-2 receptors. These peptide inhibitors can provide effective treatments for COVID-19 [18].

#### 4.6.2. Curcumin

Curcumin acts as an antiviral agent against various viral infections such as Zika virus, chikungunya virus, influenza, hepatitis and other sexually transmitted viruses. More recently, Loutfy and coworkers made curcumin-containing chitosan nanoparticles against hepatitis C virus genotype 4a. Chitosan nanoparticles have the ability to prevent 100% viral infection and proliferation in human hepatoblastoma (Huh7) cells [168]. Curcumin-loading nanoparticles show antiviral activity due to deterioration of virion membrane fluidity, but no deterioration in virion integrity. The nanoparticles were able to prevent both viral replication and entry into hepatoblastoma cells. They can be used for treating viral infections, including COVID-19 [14]. In patients with COVID-19 infection, cardiovascular symptoms are due to the imbalanced systemic inflammatory response induced by the type 1 and type 2 helper T cells [169,170]. In COVID-19 patient, curcumin has been shown to reduce inflammation in myocardial ischemia-reperfusion model by inhibiting rapid growth response-1 and reducing release of tumor necrosis factor-alpha and interleukin-6 [169,171]. This was achieved by reducing c N Jun j-terminal kinase (JNK) and NF-kB phosphorylation of nuclear displacement [169,172]. In addition, curcumin reduced the penetration of immune cells and the expression of adhesion molecules and proinflammatory mediators in vascular cells [169,173]. The abilities of curcumin to reduce cytokine release syndrome, oxidative stress, apoptosis, and tissue damage following viral infection suggest that it can be used as a promising agent for COVID-19 treatment [174].

#### 4.6.3. Dexamethasone

At the preclinical level, several various diseases such as inflammatory bowel disease, wound healing, liver fibrosis, rheumatoid arthritis, cancer, and multiple sclerosis, have been successfully treated with dexamethasone nanomedicines in the past [175,176,177].

Dexamethasone is suggested to be helpful for COVID-19 treatment based on their potential accumulation in macrophages. The pulmonary delivery of dexamethasone liposomes performed better than free dexamethasone when targeting alveolar macrophages as an approach for intervention in the subacute phase of COVID-19. This is because dexamethasone liposomes can induce an anti-inflammatory response in primary human monocytes and proinflammatory macrophages [178]. The dexamethasone loaded liposomes reduced release of proinflammatory cytokines, matrix degrading enzymes, and other signaling molecules which are involved in edema and progressive tissue damage in COVID-19 [178]. Dexamethasone nanomedicines has proven to be very useful in inhibiting fibrosis. As pulmonary fibrosis is currently a major complication of long-term control of COVID-19 [179], dexamethasone nanomedicines (inhaled or intravenous) is able to meet the urgent medical need for COVID-19 management. 

The use of dexamethasone was recommended by four senior UK medical officers on 16 June 2020 for COVID-19-positive patients with respiratory support, based on provisional data released by the RECOVERY test [180,181]. About 6000 patients in 170 NHS trusts were employed for the dexamethasone test arm. This experiment showed that dexamethasone treatment was able to reduce mortality for ventilated patients and patients receiving oxygen therapy by 35% and 20%, respectively [181,182]. It was also found that there was no improvement in patients who did not receive respiratory support. Therefore, the use of dexamethasone is recommended only in patients who are under respiratory support [181,182]. Given that dexamethasone nanomedicine could improve the survival of critically ill patients, it could be potential candidate to fight against COVID-19 [175].

#### 4.6.4. Nanoceria

Nanoceria is one of the nanomedicines for treating acute inflammation. It was found that by inhibiting NFκB signaling and lipopolysaccharide-induced MAPK signaling (Figure 4D), it reduced severe sepsis-related mortality [183,184]. Manne et al showed that nanoceria is more effective against peritonitis and has effective anti-inflammatory activity [185]. Severe inflammatory and cytokine storms in COVID-19 have significant health risk to patients. Therefore, the use of nanoceria to suppress cytokine storms and reduce general inflammation can be useful.

Nanoceria has been shown to reduce cytokine signaling by affecting a number of cytokine sources, including the paracrine and autocrine pathways, and it can directly reduce cytokine synthesis or inhibit receptor interaction of cytokines. Nanoceria is reported to block cytokine production by regulating the Nrf2/NFκB, p65-NFκB, and MAP kinase/NFκB pathways [184,186]. Hence, intervention with nanoceria can significantly inverse cytokine levels in COVID-19 patients and reduce disease progression.

Further, nanoceria has shown to have a positive impact on chronic inflammation, which would potentially be effective against neuroinflammation caused by COVID-19 [187]. Several studies showed that nanoceria improved the lifespan of brain cells and protected them from free radicals and mechanical shock [188,189,190]. In addition, the protective role of nanoceria against oxidative damage and inflammation caused by hypobaric hypoxia and oxidative stress were described [191]. This was associated with the role of nanoceria in protecting the lungs of COVID-19 patients.

## 5. Conclusions and Future Perspectives

In summary, this review presents an overview of the state-of-the-art research in nanotechnology for COVID-19 prevention, diagnostics and treatment. The exceptional properties of nanomaterials, including its strong optical and electrochemical properties, controllable sizes, biocompatibility and cost-effectiveness play a key role in a broad range of applications. Their properties can be easily tuned by modification and functionalization process using various substrates, offering tremendous potential for practical applications. Despite their significant advances, research in COVID-19 is still in the early stage and there remain many challenges. A lack of knowledge and available resources about the characteristics and aspects of COVID-19 pathophysiology as well as the mechanisms involved in the nano-biointerface remains a challenge. In addition, some nanomaterials might be capable of detecting or interacting with COVID-19 virus, preventing their action, and modulating human immune response to fight against the virus, but their multifunctional potential requires further investigations [192]. Therefore, more research that involves the in-depth study of interaction between the viral particles and nanoparticles are essential to obtain further information on the functionality, mechanisms of action and effects of nanoparticles on the virus. This information is critical in determining the optimal approaches in the prevention, diagnosis and treatment of COVID-19. 

Besides that, one of the main challenges is to ensure the safe use of nanomaterials. The behaviour changes of nanomaterials in blood circulation should be extensively studied and evaluated. The use of biodegradable nanoparticles is vital to ensure complete excretion from human body. In vivo studies should also be conducted to better understand the toxicokinetic of nanoparticles in the long term in human body. Besides that, large-scale production of high-quality nanoparticles is crucial in the response to the COVID-19 pandemic. Therefore, an effective approach for large-scale production with precise control over their size, surface modifications and other parameters at minimal cost is critical especially for producing vaccines and therapeutic agents.

Moreover, nanomaterial-based biosensors that meet ASSURED criteria: Affordable, Sensitive, Specific, User-friendly, Rapid and Robust, Equipment-free, and Deliverable to end users should be developed for rapid diagnostics of COVID-19. The integration of nanomaterials such as carbon-based nanoparticles into the detection device could generate ultrasensitive detection approaches for long-term monitoring of patient health. While improving detection sensitivity and specificity, user steps from sample preparation to signal detection should be simplified. This could potentially be achieved through integrating all operation steps into a single device. The development of simple, portable and equipment-free device would definitely be useful for COVID-19 screening at remote settings. Additionally, incorporating smartphone apps would enable tracking of patient health status for onsite health monitoring. 

In short, as the pandemic evolves, the development of simple and cost-effective nanomaterial-based products is of paramount importance for the prevention, diagnostics and treatment of COVID-19. Through research and development, nanotechnology could help preventing viral dissemination, improving diagnostics sensitivity using only a tiny amount of biological sample. The conventional therapies could also be improved through delivering antiviral nanoparticles to activate host immune response against the viruses. We envision that nanotechnology is a powerful tool to combat COVID-19 and more studies are required to contribute new scientific knowledge to boost the use of nanomaterials in managing the COVID-19 outbreak and future pandemics.

## Figures and Tables

**Table 1:** Summary of the role of nanomaterials for COVID-19.

Nanomaterial	Function	Main Role for COVID-19	Refs.
Metal nanoparticle	- induce structural changes in viral S protein, resulting in viral neutralization - modified (nucleic acid or protein bound) and integrated into sensors for COVID-19, particularly for colorimetric detection	prevention, diagnostics and treatment	[14]
Lipid nanoparticle	- serve as an mRNA vaccine by acting as a carrier to introduce mRNA into host cells - protect mRNA from degradation	prevention	[15]
Magnetic nanoparticle	- conjugated with a probe is used to detect complementary target sequence of SARS-CoVs	diagnostics	[16]
Quantum dot	- inhibit binding of S protein receptor of coronavirus to host cells - prevent viral RNA genome amplification - incorporated into sensor, acting as fluorescent label for COVID-19 detection - generate antiviral radicals by interacting with light	virus inactivation, prevention, diagnostics and treatment	[17]
Carbon-based nanoparticle	- inactivate virus and inhibit its entry into host cells - integrated into diagnostic platform for COVID-19 detection, especially for sensitive electrochemical detection	virus inactivation, prevention, diagnostics and treatment	[8]
Nanodrug	- bind to viral receptors and inactivate them - block virus from entering host cells	treatment	[18]
Nanozyme	- incorporated into sensors for chemiluminescence detection of COVID-19 viruses	diagnostics	[19]
Exosome	- target, bind and suppress cellular uptake of coronavirus - inhibit viral replication, which enables COVID-19 treatment	treatment	[20]
